# Neonatal exposure to an inflammatory cytokine, epidermal growth factor, results in the deficits of mismatch negativity in rats

**DOI:** 10.1038/s41598-019-43923-y

**Published:** 2019-05-16

**Authors:** Eiichi Jodo, Hiroyoshi Inaba, Itaru Narihara, Hidekazu Sotoyama, Eiko Kitayama, Hirooki Yabe, Hisaaki Namba, Satoshi Eifuku, Hiroyuki Nawa

**Affiliations:** 1 Department of Systems Neuroscience, Fukushima Medical University School of Medicine, Fukushima, 960-1295 Japan; 2 Department of Molecular Neurobiology, Brain Research Institute, Niigata University, Niigata, 951-8585 Japan; 3 Department of Neuropsychiatry, Fukushima Medical University School of Medicine, Fukushima, 960-1295 Japan

**Keywords:** Neurophysiology, Schizophrenia

## Abstract

Perinatal exposure to epidermal growth factor (EGF) induces various cognitive and behavioral abnormalities after maturation in non-human animals, and is used for animal models of schizophrenia. Patients with schizophrenia often display a reduction of mismatch negativity (MMN), which is a stimulus-change specific event-related brain potential. Do the EGF model animals also exhibit the MMN reduction as schizophrenic patients do? This study addressed this question to verify the pathophysiological validity of this model. Neonatal rats received repeated administration of EGF or saline and were grown until adulthood. Employing the odd-ball paradigm of distinct tone pitches, tone-evoked electroencephalogram (EEG) components were recorded from electrodes on the auditory and frontal cortices of awake rats, referencing an electrode on the frontal sinus. The amplitude of the MMN-like potential was significantly reduced in EGF-treated rats compared with saline-injected control rats. The wavelet analysis of the EEG during a near period of tone stimulation revealed that synchronization of EEG activity, especially with beta and gamma bands, was reduced in EGF-treated rats. Results suggest that animals exposed to EGF during a perinatal period serve as a promising neurodevelopmental model of schizophrenia.

## Introduction

Epidermal growth factor (EGF) is a growth factor and an inflammatory cytokine with 53 amino acid residues, and is known to be a structural homologue of neuregulin-1, whose mutation is implicated in schizophrenia^1,2^. Both EGF and neuregulin-1 play crucial roles in neuronal proliferation, differentiation and/or survival of dopamine neurons and GABAergic cells^3–5^. The receptors for EGF and neuregulin-1 (i.e. ErbB1 and ErbB4) are distributed in various regions of the brain, and can form heterodimeric complexes involved in common signaling pathways^6–8^. The functional gene mutation of EGF is found in patients with schizophrenia and/or other psychiatric diseases^9–11^, but the genetic association of EGF with these illnesses is controversial^1,2,12^. In order to investigate the neurobiological roles of EGF/ErbB1 signals in schizophrenia-related behavioral phenotypes, we attempted to develop an animal model for schizophrenia using various approaches like neonatal injection, intraventricular administration and genetic overexpression of EGF^3,13,14^. Peripheral administration of EGF or its orthologues (i.e. transforming growth factor alpha, epiregulin) in a neonatal period induces similar cognitive and behavioral abnormalities during the post-pubertal stages in mice, rats and monkeys^3,15–17^. Due to this similarity of age at onset between EGF-treated animals and those with schizophrenia, we hypothesized that the animals exposed to EGF or other ErbB1 ligands in the early stages of development may prove to be a useful neurodevelopmental model of schizophrenia^18^. However, the pathophysiological traits of the animal model remain to be elucidated.

Mismatch negativity (MMN) is an event-related potential (ERP), which is usually recorded on the human scalp with a negative peak latency of 100–200 ms after the onset of the stimulus, and is elicited by a deviant (rare and unexpected) stimulus^19–21^. Many studies have reported that the amplitude of MMN in patients with schizophrenia is reduced by comparison with healthy controls^22–26^. A recent study also reported that a reduction of MMN amplitudes in early stages of schizophrenia was associated with high plasma levels of glutamate, suggesting that the MMN be a useful biological marker of aberrant glutamatergic neurotransmission in the first episode psychosis^27^. EGF-treated animals also exhibited abnormalities in glutamatergic neurotransmission, including a reduced expression of AMPA receptors in parvalbumin-positive GABAergic neurons^28^. MMN is now considered to be a promising biological marker for schizophrenia. Non-human animals including rats also exhibit the MMN-like potential that has similar characteristics in waveform and response properties to those of the human MMN^24,29–35^. Therefore, MMN can bridge a biological gap between human and non-human animals to examine higher brain function.

In the present study, we recorded the MMN-like potential in one of the putative animal models for schizophrenia, which was perinatally EGF-treated rats, and characterized their deficits in MMN-like potential to evaluate validity of this model for schizophrenia.

## Results

### Event-related potential (ERP) waveforms recorded from electrodes on the auditory- and the frontal cortices

Perinatally EGF-treated rats as well as saline-treated rats (3–4 months old) were acclimated to the recording chamber in dark cycles (i.e., awake states) and then subjected to electroencephalogram (EEG) recording from two electrodes on the auditory- and the frontal cortices with a reference electrode on the frontal sinus. Event-related potentials were elicited with standard tones (3000 Hz, 1800 times) and deviant tones (6000 Hz, 200 times) in the oddball paradigm or with 10 different tones (1000–8000 Hz, 200 times each) in the many-standards paradigm (see details in Methods). In the many-standards paradigm the auditory stimulus that was physically the same stimulus as the deviant tone was presented among other 9 tones, which had different frequencies from each other with the same probability of occurrence (10%). Then, this stimulation paradigm allowed us to make the adaptation-independent comparison between deviant- and non-deviant stimuli^36,37^.

**Figure 1:**
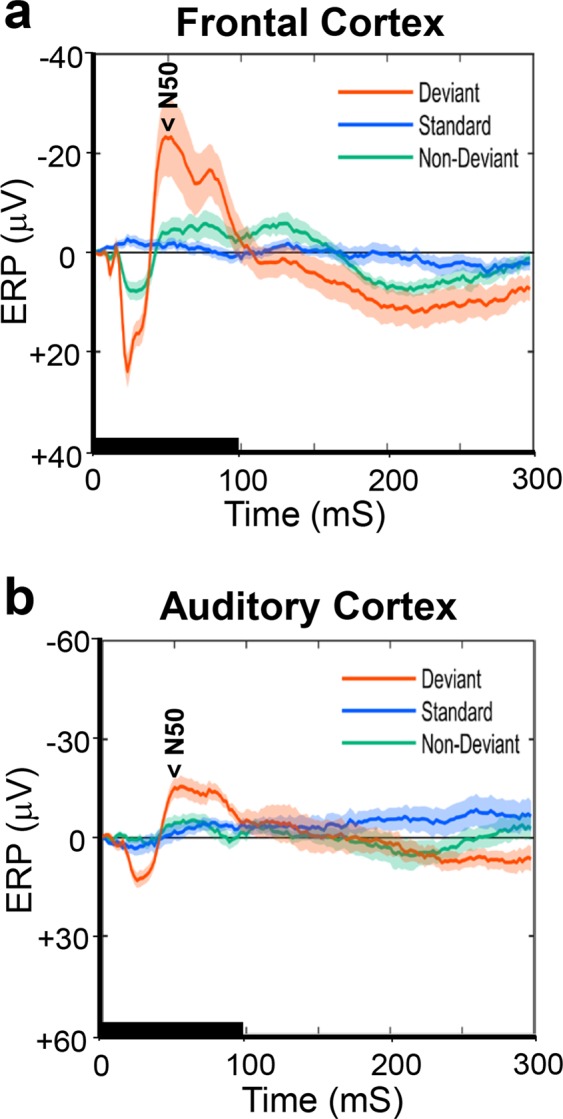
Difference in the average event-related potentials (ERPs) between control and EGF-treated rats in the frontal cortex. ERP waveforms were recorded from an electrode on the frontal cortex in control (**a**)- and EGF (**b**)- treated rats and were grand averaged across subjects. Waveforms from the 6 kHz deviant (orange), the 3 kHz standard (sky blue), and the 6 kHz non-deviant (green) tones. The non-deviant tone has the same pitch and probability of occurrence as the deviant tone but does not stand out from the other stimuli (1, 1.5, 2, 3, 4, 5, 6, 7, 7.5 and 8 kHz). The shaded area around each ERP trace denotes the mean ± standard error (SE). The tone stimulation is marked with a thick horizontal line lasting for 100 ms. The major ERP component of N50 is marked. Data were obtained from 12 control and 12 EGF-treated rats.

**Figure 2:**
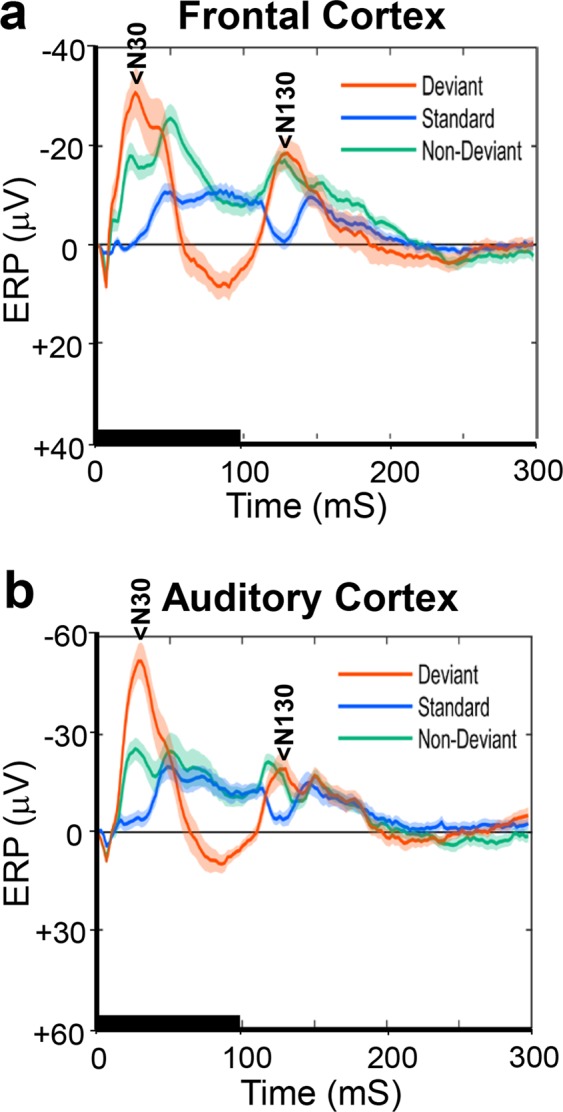
Similarity of the average ERP waveforms in the auditory cortex between control and EGF-treated rats. ERP waveforms were recorded in the auditory cortex in control (**a**)- and EGF (**b**)-treated rats, and were grand averaged across subjects. The major ERP components of N30, P80, and N130 are identified. Other descriptions are the same as those depicted in Fig. 1.

Grand average ERP waveforms to deviant, standard, and non-deviant tones are presented in Fig. 1 (frontal cortex) and Fig. 2 (auditory cortex). Consistent with previous reports^36,38^, in EEG recording from an electrode on the frontal cortex (FC), a human-MMN-like negative potential was elicited around 50 ms after the start of deviant tone presentation. Then, there was a sharp positive potential that peaked at around 20 ms (Fig. 1a). However, no clear potential component was elicited by the standard tone. By contrast, in EEG recording from an electrode on the auditory cortex (AC) there were two separate negative potentials which peaked at around 30 ms and 130 ms, respectively (Fig. 2a). No notable negative potential component was elicited in the temporal range where the MMN-like potential peaking around 50 ms was detected in the FC. However, a small positive component peaking around 80 ms appeared between the large two negative components. The negative components in the auditory cortex appear to respond to either the leading or the falling edge of the tone. Since the waveform between the FC and the AC was considerably different, ERP components derived from these two locations were independently measured using different criteria. The MMN-like component in the FC was defined as the maximum negative peak in the temporal range of 40–140 ms (N50) after the start of tone presentation, in the AC of 10–60 ms (N30), 40–140 ms (P80), and 100–160 ms (N130).

**Figure 3:**
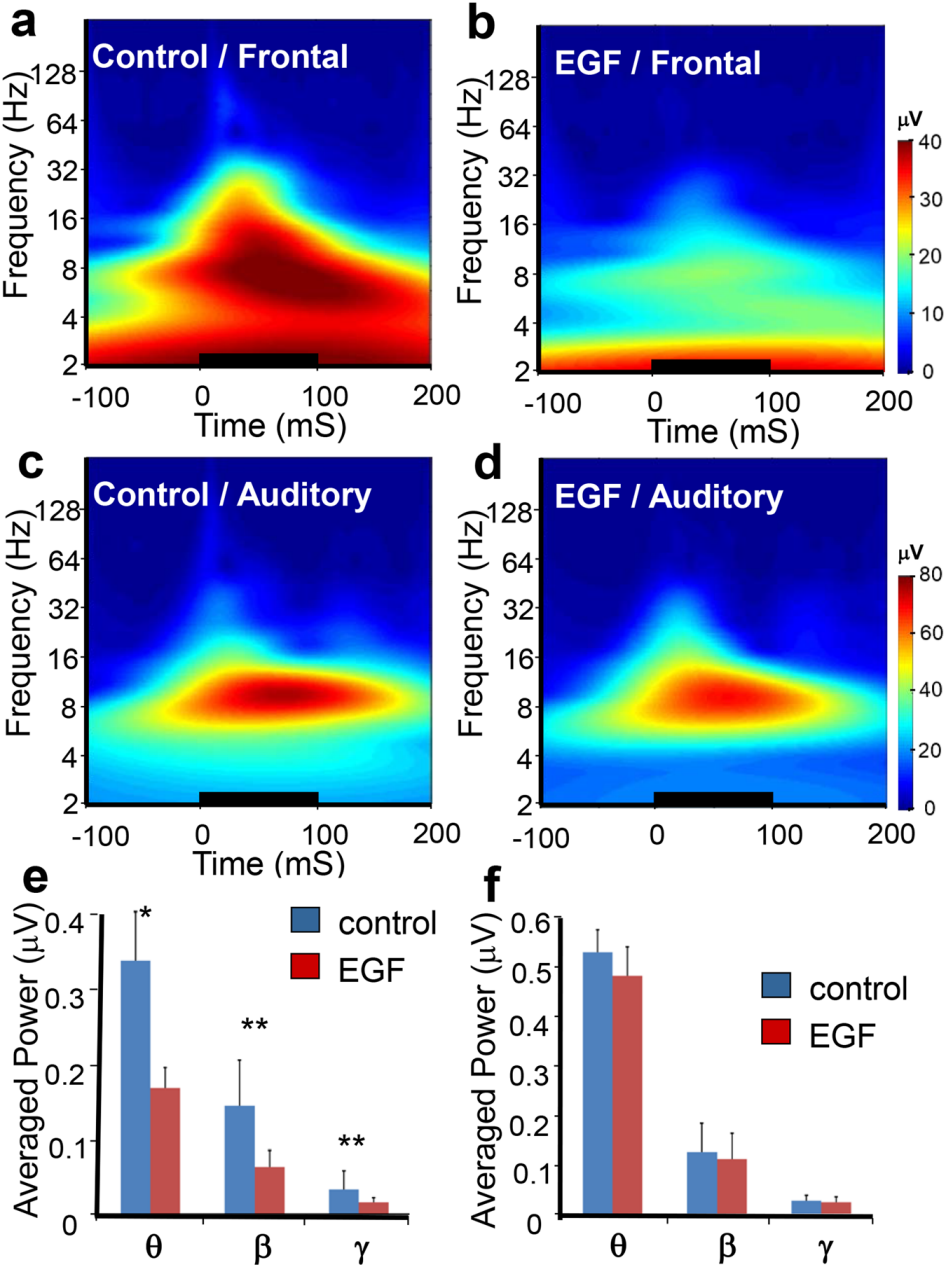
Grand average time-frequency responses of EEG activity from the deviant tone. The deviant tone-evoked EEG responses in the frontal cortex **(a**,**b**,**e**) and auditory cortex (**c**,**d**,**f**) of control (**a**,**c**) and EGF-treated (**b**,**d**) rats that were subjected to Wavelet analysis. Data was transformed into grand average time-frequency responses. Averaged magnitude responses (μV, from 25 ms to 140 ms) in individual frequency ranges (theta 4–13 Hz, beta 14–29 Hz, gamma 30–80 Hz) were compared in the frontal cortex (**e**) and auditory cortex **(f**) between control and EGF-treated rats. The tone stimulation is marked with a thick horizontal line starting at 0 ms and lasting for 100 ms. Asterisks above the bars denote a statistically significant difference between groups (*p < 0.05, **p < 0.01).

### Effects of stimulus conditions on the amplitude of ERP components

In order to examine effects of neonatal EGF-exposure and stimulus conditions on each ERP component (N50 recorded at the FC, N30, P80, and N130 recorded at the AC), repeated measures analysis of variance (rANOVA) was performed on the amplitude data for the following two factors, the groups (EGF vs. control), stimulus type (deviant, standard, non-deviant). The non-deviant tone presented in the many-standards paradigm had the same physical property and probability of occurrence as did the deviant tone, but it was not deviant. The results of rANOVA showed that in the N50 component there were significant main effects of groups (F(1, 21) = 188.83, p < 0.001) and stimulus type (F(2, 20) = 46.51, p < 0.001), and a significant interaction between groups and stimulus type (F(2, 20) = 5.14, p < 0.017). The significant group effect indicates that the N50 in the EGF group (−10.41 ± 2.20 μV) was significantly smaller than the one in the control (−17.72 ± 2.10 μV). Post hoc analyses exhibited that the N50 to the deviant tone (−26.72 ± 3.61 μV) was significantly larger (p < 0.001) than the ones to both the standard (−5.66 ± 0.71 μV) and the non-deviant tones (−9.80 ± 1.17 μV), and the N50 to the non-deviant tone was significantly larger than the one to the standard tone (p < 0.003). These results indicate that the N50 component may have the deviance-specific characteristic as the human MMN does. They further revealed that the difference in the N50 amplitude between groups (control vs. EGF) was statistically significant only to the deviant tone (control: −36.25 ± 4.99 μV, EGF: −17.20 ± 5.21 μV, F(1, 21) = 6.97, p < 0.05), and no significant group difference was detected to the standard- (control: −4.72 ± 0.98 μV, EGF: −6.59 ± 1.02 μV, F(1, 21) = 1.75, p < 0.6) and the non-deviant tones (control: −12.17 ± 1.62 μV, EGF: −7.43 ± 1.69 μV, F(1, 21) = 4.12, p < 0.11).

The N30 component recorded from the AC exhibited no significant main effect of groups (control: −38.24 ± 4.44 μV, EGF: −38.14 ± 4.63 μV, F(1, 21) = 0.00, p < 0.99) nor interaction between groups and stimulus type (F(2, 20) = 3.49, p < 0.06). This result indicates that the amplitude N30 did not differ between EGF- and control groups, regardless of stimulus type (control: −53.80 ± 7.20 μV, EGF: −58.84 ± 7.52 μV for deviants, control −18.42 ± 3.28 μV, EGF −21.70 ± 3.42 μV for standards, and control: −42.50 ± 4.59 μV, EGF: −33.86 ± 4.80 μV for non-deviants). The N30, however, exhibited a significant main effect of stimulus type (F(2, 20) = 37.58, p < 0.001). Post hoc analyses revealed that the N30 to the deviant tone (−56.32 ± 5.21 μV) was significantly larger (p < 0.001) than the ones to the standard- (−20.06 ± 2.37 μV) and non-deviant (−38.18 ± 3.32 μV) tones, and the N30 to the deviant tone was significantly larger than the one to the standard tone (p < 0.001). The N130 component recorded from the AC also exhibited no significant main effect of groups (control: −27.22 ± 2.55 μV, EGF: −23.02 ± 2.67 μV, F(1, 21) = 1.29, p < 0.27) nor interaction between groups and stimulus type (control: −33.52 ± 4.54 μV, EGF: −25.76 ± 4.74 μV for deviants, control: −19.04 ± 2.59 μV, EGF: −18.61 ± 2.70 μV for standards, and control: −29.09 ± 2.71 μV, EGF: −24.69 ± 2.83 μV for non-deviants, F(2, 20) = 0.826, p < 0.45). A significant main effect of stimulus type was detected in the N130 (F(2, 20) = 10.89, p < 0.002). Post hoc analyses revealed that the N130 to the standard tone (−18.82 ± 1.87 μV) was significantly smaller (p < 0.013) than the ones to both the deviant- (−29.64 ± 3.28 μV) and non-deviant (−26.89 ± 1.96 μV) tones, while no significant difference was detected between the latter two tones (p < 1.00). In the P80 component recorded from the AC statistical analyses showed a significant main effect of stimulus type (F(2, 20) = 30.48, p < 0.001). Post hoc analyses revealed that the P80 to the deviant tone (17.04 ± 2.69 μV) was significantly larger (p < 0.001) than the ones to both the standard- (0.86 ± 1.44 μV) and the non-deviant (−4.36 ± 1.78 μV) tones, and the P80 to the standard tone was significantly larger than the one to the non-deviant tone (p < 0.05). In the P80 component there were no significant group effect (control: 4.23 ± 2.04 μV, EGF: 4.79 ± 2.14 μV, F(1, 21) = 1.81, p < 0.852) nor interaction (control: 17.11 ± 3.72 μV, EGF: 16.98 ± 3.89 μV for deviants, control: 2.93 ± 1.98 μV, EGF −1.22 ± 2.07 μV for standards and, control: −7.34 ± 2.46 μV, EGF −1.38 ± 2.57 μV for non-deviants, F(2, 20) = 3.09, p < 0.069).

In additional rats we examined repetition effects of 6000 Hz tone on ERP components. The grand average ERP waveforms recorded from the FC and the AC are presented in Supplementary Figures S1 and S2, respectively. The rANOVA was performed on the amplitude data of N50, N30, P80, N130 components for the following two factors, the groups (EGF vs. control), stimulus type (deviant, standard, non-deviant). The results of rANOVA showed that in the N50 component derived from the FC there was a significant main effect of stimulus type (F(2, 13) = 30.08, p < 0.001). Post hoc analyses revealed that the amplitudes of N50 to deviant- and non-deviant tones were significantly larger than those to the standard tones, and the N50 to the nondeviant tone was significantly smaller than the one to the deviant tone (deviant: −50.00 ± 5.62 μV, non-deviant: −24.30 ± 1.84 μV, standard: −14.68 ± 2.26 μV, deviant vs. non-deviant: F(1, 14) = 16.40, p < 0.005, non-deviant vs. standard: F(1, 14) = 8.81, p < 0.032). This result indicates the repetition of the same tone can affect the amplitude of N50 (repetition effect), which is one of the necessary property for being identified as a MMN in animal models, as well as being larger to the deviant tone than to the non-deviant one (deviant effect)^36,37^. A significant main effect of group was again detected (Control: −34.73 ± 3.32 μV, EGF: −24.59 ± 2.93 μV, F(1, 14) = 5.25, p < 0.039). No significant interaction between group and stimulus type was detected (F(2, 13) = 1.745, p < 0.214).

In the N30 component derived from the AC there were a significant main effect of stimulus type (F(2, 13) = 20.04, p < 0.001) and a significant interaction between group and stimulus type (F(2, 13) = 6.77, p < 0.02). Post hoc analyses showed that the stimulus type did not have a significant effect on the N30 amplitude in the control group (deviant: −45.89 ± 5.31 μV, non-deviant: −46.34 ± 7.03 μV, standard: −33.48 ± 4.99 μV, F(2, 5) = 4.56, p < 0.076), while it was significantly changed depending on the stimulus type in the EGF group (deviant: −65.62 ± 4.01 μV, non-deviant: −40.95 ± 2.99 μV, standard: −27.84 ± 2.83 μV, deviant vs. non-deviant: F(1, 8) = 69.88, p < 0.001, non-deviant vs. standard: F(1, 8) = 8.47, p < 0.06). The N30 exhibited no other significant group difference (control: −41.90 ± 3.49 μV, EGF: −44.80 ± 3.08 μV, F(1, 14) = 0.389, p < 0.544). In the P80 component from the AC only a significant main effect of stimulus type was detected (F(2, 13) = 16.78, p < 0.001). Post hoc analyses showed that the P80 to the standard tone was significantly smaller than the ones to the deviant and the non-deviant tones (deviant: 43.01 ± 7.51 μV, non-deviant: 3.31 ± 2.10 μV, standard: 1.42 ± 2.60 μV, deviant vs. non-deviant: F(1, 14) = 36.13, p < 0.001, non-deviant vs. standard: F(1, 14) = 2.42, p < 0.428). There were no significant group difference (control: 10.10 ± 4.40 μV, EGF: 17.31 ± 3.88 μV, F(1, 14) = 1.51, p < 0.25) and interaction (F(2, 13) = 0.02. p < 0.980). The N130 component from the AC was also significantly affected only by the stimulus type (F(2, 13) = 7.75, p < 0.007). Post hoc analyses revealed that the N130 to the non-standard tone was significantly larger than the ones to the deviant and the standard tones (deviant: −22.21 ± 4.58 μV, non-deviant: −37.90 ± 2.46 μV, standard: −29.47 ± 2.76 μV, deviant vs. non-deviant: F(1, 14) = 16.25, p < 0.005, non-deviant vs. standard: F(1, 14) = 8.04, p < 0.041). There were no significant group difference (control: −35.65 ± 4.16 μV, EGF: −24.08 ± 3.67 μV, F(1, 14) = 4.35, p < 0.057) and interaction (F(2, 13) = 0.60. p < 0.565), though the group difference was statistically marginal.

### Time-frequency characteristics of EEG activity to the deviant tone

To analyze the time-frequency characteristics of EEG activity to the deviant tone that exhibited the marked difference between EGF and control rats, we performed wavelet analysis on EEG data 100 ms before and 200 ms after each tone was presented. The average magnitude (the modulus of a complex number; the square root of power) for each frequency over time across subjects is presented in Fig. 3. In EEG recording from an electrode on the FC, the magnitude distribution pattern after the start of the deviant tone appears markedly different between the two groups (EGF vs. control) (Fig. 3a,b). However, no such a difference was manifested in recording from the AC (Fig. 3c,d). Since the EEG activity with frequency range from the theta wave to the gamma wave appears particularly high in the period temporarily overlapping with the development of the MMN component, we calculated the average values of the EEG magnitude from 24–140 ms after the start of the tone in each frequency band (θ: 4–13 Hz; β: 14–29 Hz; γ: 30–80 Hz) as representative values of magnitude in each subject (Fig. 3e,f). Repeated measures analysis of variance (rANOVA) was performed on the magnitude data for the following 3 factors, the groups (EGF vs. control), electrode location (FC vs. AC) and frequency band (θ, β, γ). The results of rANOVAs showed that the average magnitude over the three frequency bands was significantly smaller in the EGF group compared with the control group (F[1, 22] = 7.278, *p* < *0.013*). The group difference in the EEG magnitude differed depending on the recorded location and the frequency band (F[2, 21] = 3.872, *p* < *0.037*). Post-hoc analyses revealed that the significant reduction of EEG magnitude in the EGF group was detected in the theta, beta and gamma bands recorded from the FC (θ: F[1, 22] = 6.840, p < 0.05, β: F[1, 22] = 11.125, *p* < *0.01*, γ: F[1, 22] = 19.479, p < 0.01; see Fig. 3a,b,e), while a significant group difference could not be detected in the EEG magnitude recorded from the AC (θ: F[1, 22] = 0.359, p < 0.555, β: F[1, 22] = 0.926, p < 0.346, γ: F[1, 22] = 0.873, p < 0.360; see Fig. 3c,d,f).

In order to examine a possible difference between groups (EGF vs. control) in the magnitude of three EEG components (θ, β, γ) during a baseline period of 100 ms before each tone-presentation we performed rANOVA on the average magnitudes of the EEG components for the 100 ms baseline period in a similar manner to that mentioned above. The results showed that the significant main effect of groups was not detected (F(1, 22) = 1.977, p < 0.175), but there was a significant interaction among electrode location, frequency band, and groups (F(1, 21) = 4.844, p < 0.02). Post hoc analyses revealed that only the magnitude of beta-band EEG derived from the FC was significantly smaller in the EGF group than the one in the control group (θ: F(1, 22) = 4.995, p > 0.05, β: F(1, 22) = 9.691, p < 0.05; γ: F(1, 22) = 0.06, p < 0.938 for FC, and θ: F(1, 22) = 0.163, p < 0.700, β: F(1, 22) = 0.318, p < 0.580, γ: F(1, 22) = 0.609, p < 0.450 for AC). Mean values of the baseline magnitude in each frequency band are presented in the Supplementary Figure S3. Because the baseline magnitude was significantly different in the beta band between groups, we calculated the relative magnitude that was the ratio of the EEG magnitude from 24–140 ms after the start of the tone in each frequency band to the baseline EEG magnitude in each band to cancel the baseline difference. Mean values of the relative magnitude in each frequency band are presented in the Supplementary Figure S4. The rANOVA was also performed on the relative EEG magnitude in a similar manner to that for the EEG magnitude. The results of rANOVA showed that there were a significant main effect of groups (F(1, 22) = 9.559, p < 0.006) and a significant interaction among groups, electrode location, and frequency band (F(2, 21) = 4.780, p < 0.020). Post hoc analyses revealed that only the relative EEG magnitude derived from the FC in the gamma band was significantly smaller in the EGF groups than the one in the control group (θ: F(1, 22) = 4.417, p < 0.10, β: F(1, 22) = 3.148, p < 0.090; γ: F(1, 22) = 11.866., p < 0.02 for FC, and AC θ: F(1, 22) = 1.125, p < 0.31, β: F(1, 22) = 7.149, p < 0.10, γ: F(1, 22) = 0.019, p < 0.90 for AC).

Next, we examined a possible difference in the magnitude of EEG activity between deviant- and non-deviant tones, which were physically the same. The rANOVA was performed on the magnitude data for the following 4 factors, the groups (EGF vs. control), electrode location (FC vs. AC), stimulus type (deviant vs. non-deviant), and frequency band (θ, β, γ). The rANOVA results showed that there were significant main effects of groups (control: 14.15 ± 0.96 μV, EGF: 10.77 ± 1.00 μV, F(1, 21) = 5.96, p < 0.03), stimulus type (deviant: 17.14 ± 1.07 μV, non-deviant: 7.78 ± 0.44 μV, F(1, 21) = 118.18, p < 0.001), electriode location (FC: 8.81 ± 0.85 μV, AC: 16.11 ± 1.12 μV, F(1, 21) = 26.78, p < 0.001), and frequency band (θ: 26.79 ± 1.62 μV, β: 8.51 ± 0.56 μV, γ: 2.09 ± 0.14 μV, F(2, 20) = 144.02, p < 0.001). There were also significant interactions between groups and stimulus type (F(1, 21) = 4.51, p < 0.05), and among groups, stimulus type, and electrode location (F(1, 21) = 4.63, p < 0.05). Post hoc analyses revealed that a significant group difference was detected only in EEG activity recorded from the FC to the deviant tone (control_FC_deviant: 17.32 ± 2.08 μV, EGF_FC_deviant: 8.60 ± 2.17 μV, F(3, 20) = 4.57, p < 0.015, control_FC_non-deviant: 5.38 ± 0.42 μV, EGF_FC:_non-deviant 3.94 ± 0.44 μV, F(3, 19) = 2.57, p < 0.09, control_AC_deviant: 22.18 ± 2.10 μV, EGF_AC_deviant: 20.47 ± 2.19 μV, F(3, 20) = 0.31, p < 0.82, control_AC_non-deviant: 11.74 ± 1.17 μV, EGF_AC_non-deviant: 10.07 ± 1.22 μV, F(3, 19) = 1.53, p < 0.24). Another significant interaction was detected among groups, stimulus type, and frequency band (F(2, 20) = 4.27, p < 0.03). Post hoc analyses showed that the magnitude of EEG activity in the control group was significantly larger to the deviant tone in all of three bands (θ, β, γ) than the one in the EGF group (control: 42.75 ± 3.40 μV, EGF: 32.50 ± 3.55 μV, F(2, 21) = 3.67, p < 0.05 for θ, control: 13.43 ± 1.16 μV, EGF: 9.04 ± 1.21 μV, F(2, 21) = 5.40, p < 0.015, for β, and control: 3.07 ± 0.28 μV, EGF: 2.07 ± 0.29 μV, F(2, 21) = 5.41, p < 0.015 for γ). To the non-deviant tone, however, a significant group difference was detected only in the γ band (control: 18.26 ± 1.64 μV, EGF: 13.64 ± 1.71 μV, F(2, 20) = 2.48, p < 0.11 for θ, control: 5.79 ± 0.52 μV, EGF: 5.77 ± 0.54 μV, F(2, 20) = 3.13, p < 0.07 for β, and control: 1.63 ± 0.14 μV, EGF: 1.59 ± 0.14 μV, F(2, 20) = 4.04, p < 0.04 for γ).

### Phase consistency of EEG responses across trials to tone stimulations

**Figure 4:**
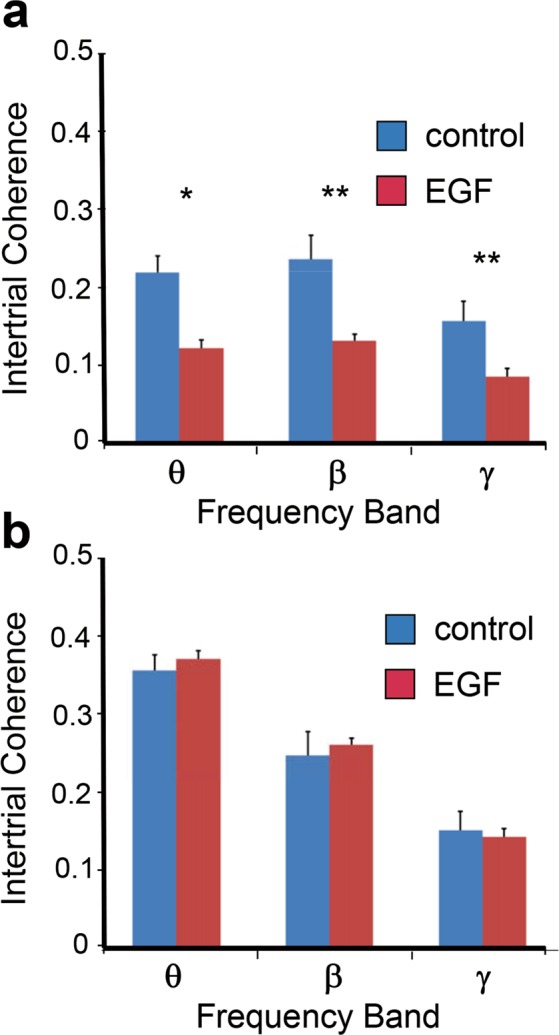
Reduced inter-trial coherences of EEG activity in the frontal cortex of EGF-treated rats to the deviant tone. Grand average inter-trial coherences (from 25–140 ms) in individual frequency ranges (theta 4–13 Hz, beta 14–29 Hz, gamma 30–80 Hz) were compared in the frontal cortex (**a**) or in the auditory cortex (**b**) between control and EGF-treated rats. Asterisks denote a significant difference between groups (*p < 0.05, **p < 0.01).

Next, we calculated an inter-trial coherence (ITC), which is a marker of phase consistency of EEG across trials independent of its power. ITC can assess ‘the temporal and spectral synchronization within EEG, the extent to which underlying phase-locking occurs’^39^ ^(p.1461)^. The ITC was calculated using Scilab (ver.6.0) and its Wavelet Toolbox, according to previous studies^39,40^. Using the ITC, the possible difference in synchronization of neural activity to auditory stimulation between groups was examined. Average values of ITC in each frequency band (θ, β, γ) during the period from 25–140 ms after the start of tone, was calculated as representative values of ITC in each subject. The mean ITCs in each frequency across subjects are presented in Fig. 4. The rANOVA was also performed on the ITC data in the same manner as it was for EEG magnitude. The results of rANOVA showed a significant main effect of groups (control 0.268 ± 0.017, EGF 0.219 ± 0.017; F(1, 22) = 4.36, p < 0.05;). Further, there was a significant interaction between group and electrode location (F[1, 22] = 24.42, p < 0.001), indicating that the ITC derived from the FC was smaller in the EGF group than in the control (F(1, 22) = 17.64, p < 0.002), but the one from the AC did not exhibit such a significant group difference (F(1, 22) = 0.089, p < 0.768). Post-hoc analyses revealed that the mean values of ITC in theta, beta and gamma bands in the FC were all significantly lower in the EGF group compared with those in the control group (F[1, 22] = 9.38, p < 0.05 for θ, F[1, 22] = 14.77, p < 0.01 for β, and F[1, 22] = 17.80, p < 0.01 for γ). None of EEG components (θ, β, γ) recorded from the AC exhibited significant group differences in the ITC (F[1, 22] = 0.138, p < 0.714 for θ, F[1, 22] = 0.298, p < 0.591 for β, and F[1, 22] = 0.221, p < 0.643 for γ).

To test a possible difference in ITC between deviant- and non-deviant tones rANOVA was performed on the ITC data for factors of groups (EGF vs. control), electrode location (FC vs. AC), stimulus type (deviant vs. non-deviant), and frequency band (θ, β, γ). The rANOVA results showed that there were significant main effects of stimulus type (F(1, 21) = 89.83, p < 0.001; deviant 0.243 ± 0.012, non-deviant 0.149 ± 0.006), and frequency band (θ: 0.245 ± 0.012, β: 0.211 ± 0.10, γ: 0.133 ± 0.005, F(2, 20) = 79.46, p < 0.001). Post hoc analyses revealed that the ITC in the theta band was significantly higher than the ones in both beta (p < 0.02) and gamma bands (p < 0.001), and the ITC in the beta band was significantly higher than the one in the gamma band (p < 0.001). There were also significant interactions between groups and stimulus type (F(1, 21) = 4.45, p < 0.05), among groups, stimulus type, and electrode location (F(1, 21) = 7.57, p < 0.02), among groups, stimulus type, and frequency band (F(2, 20) = 3.85, p < 0.04). Post hoc analyses revealed that the ITC derived from the FC in the EGF group was significantly lower than the one in the control, regardless of the stimulus type (control_deviant: 0.239 ± 0.018, EGF_deviant: 0.134 ± 0.019, F(3, 20) = 6.42, p < 0.004; control_non-deviant: 0.102 ± 0.007, EGF_non-deviant: 0.078 ± 0.007, F(3, 19) = 4.53, p < 0.02). The ITC derived from the AC, however, exhibited a significant group difference only to the non-deviant tone (control_deviant: 0.296 ± 0.019, EGF_deviant: 0.305 ± 0.020, F(3, 20) = 0.65, p < 0.6, control_non-deviant: 0.202 ± 0.015, EGF_non-deviant: 0.214 ± 0.015, F(3, 19) = 3.60, p < 0.04). Statistical analyses further showed that the ITC derived from the FC in the control group was significantly higher in all three bands than the one in the EGF group (control_θ: 0.178 ± 0.015, EGF_θ: 0.113 ± 0.016, F(2, 20) = 4.27, p < 0.03, control_β: 0.198 ± 0.014, EGF_β: 0.123 ± 0.015, F(2, 20) = 6.65, p < 0.007, control_γ: 0.134 ± 0.008, EGF_γ: 0.083 ± 0.008, F(2, 20) = 10.48, p < 0.002). The ITC derived from the AC exhibited a significant group difference only in the beta band, though its relationship was opposite to that obtained in the FC (control_θ: 0.355 ± 0.028, EGF_θ: 0.336 ± 0.029, F(2, 20) = 1.74, p < 0.21, control_β: 0.237 ± 0.019, EGF_β: 0.284 ± 0.020, F(2, 20) = 3.62, p < 0.05, control_γ: 0.156 ± 0.012, EGF_γ: 0.159 ± 0.012, F(2, 20) = 3.27, p < 0.06).

Then, we calculated the Pearson’s product-moment correlation coefficient (*r*) to examine relationship between the N50 amplitude to the deviant tone and the ITC in each frequency band (θ, β, γ). The *r* values were all statistically significant in the control group (θ: *r* = −0.769, p < 0.003, β: *r* = −0.893, p < 0.001, γ: *r* = −0.805, p < 0.002), while no significant *r* values were detected in the EGF group (θ: *r* = −0.335, p < 0.288, β: *r* = −0.117, p < 0.717, γ: *r* = −0.039, p < 0.903). The *r* values between the N50 and the average value of ITCs over three frequency bands were also calculated in each group (control: *r* = −0.939, p < 0.001, EGF: *r* = −0.090, p < 0.780). These results indicate that the synchronization of EEG components was closely related with a variation of the N50 amplitude in the control group, but not in the EGF group.

### Similarity of EEG temporal-frequency response pattern between groups

**Figure 5:**
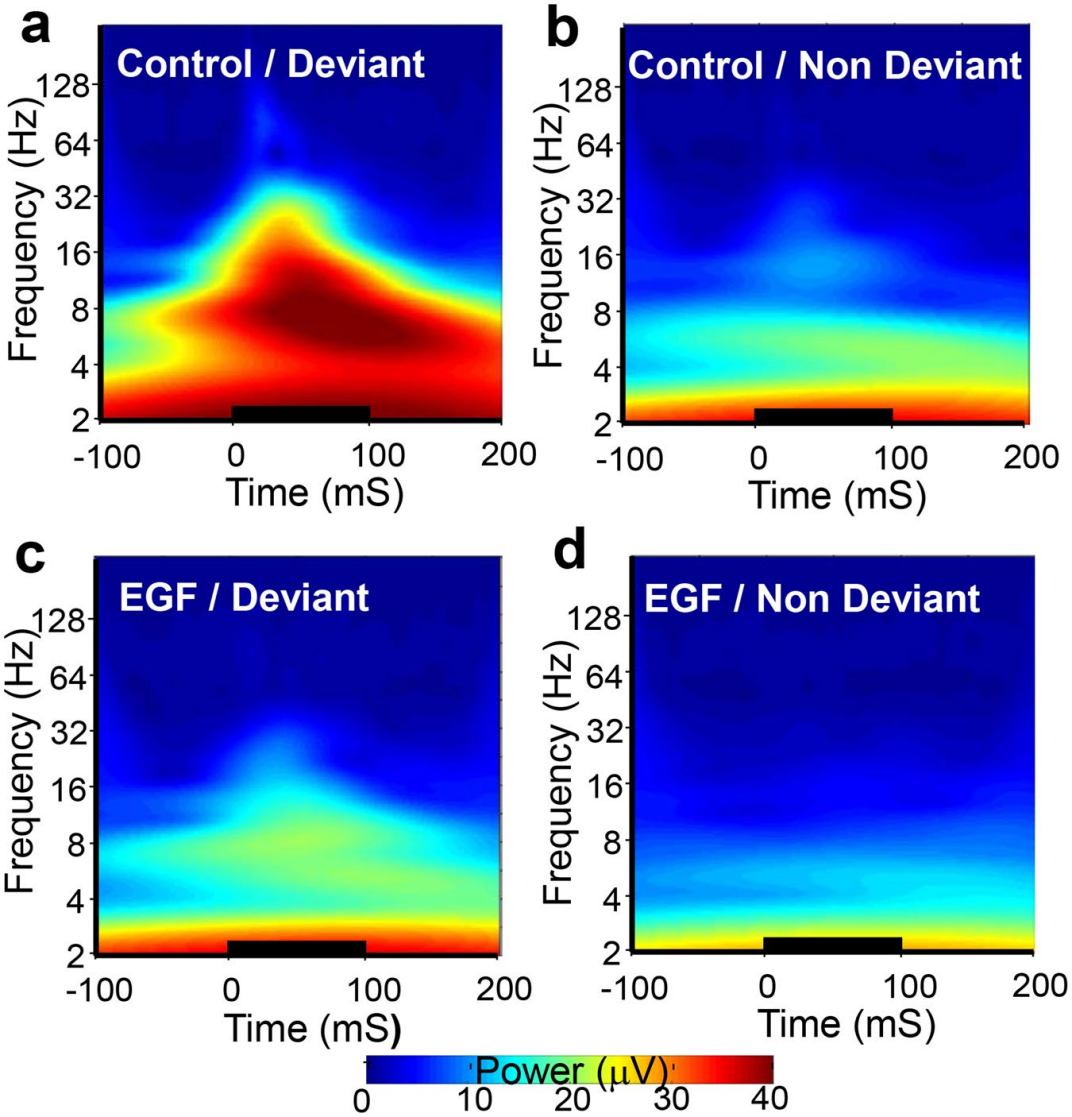
Grand average time-frequency responses of EEG activity to the deviant tone and the non-deviant tone in the frontal cortex. The deviant tone-evoked (**a**,**c**) and non-deviant tone-evoked (**b**,**d**) EEG responses of control (**a**,**b**) and EGF-treated (**c**,**d**) rats were subjected to wavelet analysis and then transformed into grand avera ge time-frequency responses. Note: There was pattern similarity between **b** and **c**. The tone stimulation is marked with a thick horizontal line starting at 0 ms and lasting for 100 ms.

To explore the EEG difference of EGF-treated rats, we analyzed and compared the time-frequency responses of FC-derived EEG activity to deviant and non-deviant tones were compared between EGF (Fig. 5c,d) and control groups (Fig. 5a,b). The temporal-frequency response pattern from the deviant tone in the EGF group (Fig. 5c) appears to be similar to that from the non-deviant tone in the control group (Fig. 5b).

In order to verify the similarity of the response pattern quantitatively, a mean value of the structural similarity index (MSSIM) was calculated^41^. It is now a popular measure for objective assessment of perceptual image quality. The MSSIM has a decimal value between −1 to +1, and is exactly 1 only when two images are identical. We set two reference images, the grand average time-frequency response image to the deviant tone (Ref-con/dev; i.e., Fig. 5a) and the one to the non-deviant tone (Ref-con/non; i.e., Fig. 5b) across subjects in the control group. A test image was the time-frequency response image in each trial to the deviant tone in the EGF group. The MSSIM between reference- and test images were calculated in each trial using MATLAB (R2017b). An average value of each single MSSIM from a subject was defined as a representative value of MSSIM in each subject.

The statistical test (t-test) revealed that the average MSSIM in magnitude between the Ref-con/non and the test image was significantly greater compared with the MSSIM between the Ref-con/dev and the test image (deviant vs. non-deviant: 0.0020 ± 0.0002, deviant vs. deviant: 0.0011 ± 0.0000, t = −4.27, d.f. = 10, p < 0.002). This result suggests that the response pattern from the deviant tone in the EGF group would be more similar to the non-deviant tone in the control group.

In contrast, the time-frequency response pattern of the ITC to the deviant tone in the EGF group appeared more similar to that in the control group (Figure not shown). The average MSSIM in ITC between the Ref-dev and the test image was significantly larger compared with the MSSIM between the Ref-non and the test image (deviant vs. deviant 0.7585 ± 0.0165, deviant vs. non-deviant 0.6832 ± 0.0230, t = 3.08, p < 0.012). These response patterns were shown also in MSSIM data derived from the auditory cortex (deviant vs. deviant: 0.0008 ± 0.0000, deviant vs. non-deviant: 0.0010 ± 0.0000; t = −9.59, p < 0.0001 for magnitude, and deviant vs. deviant: 0.7074 ± 0.0110, deviant vs. non-deviant: 0.6399 ± 0.0112; t = 5.90, p < 0.001 for ITC).

## Discussion

EEG recording from the surface electrode of the AC revealed that neither ERP amplitudes nor waveforms to the standard tone differed between groups (EGF vs. control). These results presumably ruled out the fundamental deficits in the hearing ability of this animal model and established the minimum authenticity of the present MMN experiments. EEG recording with the oddball and many-standards paradigms of different pitches obtained the following four findings: first, neonatal administration of EGF resulted in reduction of the MMN-like potential recorded at the FC of adult rats; second, gamma-band phase synchronization of EEG recorded at the FC was perturbed in EGF-treated rats; third, the MMN-like potential showed similar waveform and response characteristics to the human MMN, but it was not recorded from the surface of the AC. Finally, in the EGF-treated rats the time-frequency response patterns of FC-derived EEG magnitudes and ITC compared with the deviant tone resembled those to the non-deviant tone in the control rats, respectively. These results indicate that the EGF model represents the human MMN-like deficit that presumably involves gamma band de-synchronization. However, it remains to be explored why the MMN-like deficit was detected from the electrode on the FC, but not detected from the electrode on the AC when we recorded in the rat awake condition of dark cycle with the electrode position (i.e., primary AC and FC for recording, and the frontal sinus for reference).

In the current study, the MMN-like negative deflection to the deviant tone was recorded from the FC. This negative deflection had morphology similar to the human MMN^21^, and its amplitude in the oddball paradigm was significantly larger than in the non-deviant tone in the many standards paradigm. The non-deviant tone had the same physical properties (frequency, intensity, probability of occurrence) as the deviant one in the oddball paradigm. The non-deviant tone presented in the many-standards condition is considered to be not deviant, because all 10 different tones randomly occurred with the same probability (10%) in that condition. Further, this negative deflection to the standard tone exhibited smaller amplitude than the one to the non-deviant tone that was physically the same as the standard tone except the probability of occurrence, indicating the existence of a repetition effect on the deflection. This indicates that the MMN-like negative deflection recorded in the current study should be a change-specific ERP component that has homologous properties to the human MMN. We will refer to this human MMM-like potential as the rat-MMN hereafter.

Our present data showed that the rat-MMN component derived from the FC was significantly smaller in the EGF-treated rats compared with the control rats. The time-frequency analysis of EEG activity exhibited that the magnitude and phase consistency of EEG activity in the EGF group were both smaller in three frequency bands (theta, beta, and gamma) in timing of the rat-MMN appearance, compared with those in the control group. Such dysfunctional oscillatory activity in beta- and gamma bands has also been reported by many previous studies for patients with schizophrenia^42–47^. Further, a recent study reported that the magnitude and phase consistency of theta activity to the deviant tone were also reduced in patients with schizophrenia^48^. In our current study, however, the relative EEG magnitude calculated for correcting baseline difference between groups exhibited a significant group difference only in the gamma band. This fact suggests that a disturbance in the gamma EEG activity may primarily contribute to reduced MMN-like potential in the EGF-treated rats. Then, what is the reason why the rat-MMN was markedly smaller in the EGF group? Due to the phase consistency of the FC-derived EEG across the trials exhibiting a reduction in the EGF-treated rats during the development of rat-MMN, reduction of the rat-MMN may partly be produced by a reduction of synchronization in EEG activity induced to each deviant tone. This hypothesis may further be supported by the result that the ITC value, a marker of phase consistency of EEG across trials, in the control group was highly correlated with the EEG magnitude of the rat-MMN, especially in beta- and gamma bands (*r* > 0.8), but not in the EGF group (*r* < 0.2). In the EGF-treated rats the level of EEG synchronization dissociated from amplitude variation of the rat-MMN, unlike in the case of the control group, possibly because fluctuation in low level of EEG-synchronization could little contribute to variation in the amplitude of rat-MMN. Our previous study showed that systemic administration of EGF to mouse neonates reduced the expression of glutamic acid decarboxylases (GAD65 and GAD67) and parvalbumin^5^ in the FC, indicating possible dysfunction of GABAergic neurons in the EGF-treated animals. Because activity of GABAergic interneurons is implicated in the generation of γ oscillation of EEG activity^49–51^, the reduced phase consistency of EEG activity of this model rats is likely to associate with their deteriorated GABAergic system.

A likelihood of adaptation to auditory stimuli is another possible factor contributing to the reduced rat-MMN in the EGF group. If the reduction of rat-MMN stems from higher adaptation potential of EGF-treated rats, the amplitude of rat-MMN to the deviant tone should almost be equal to the one to the non-deviant tone. Our present data, however, exhibited a significant difference in the rat-MMN amplitude between deviant- and non-deviant tones, and does not support this adaptation hypothesis. Therefore, our result indicates that neonatal administration of EGF may induce a dysfunction in the neural circuits involved in generating the MMN.

However, ERP components derived from the auditory cortex did not significantly differ between the groups (EGF vs. control), and between deviant- and non-deviant tones. None of AC-derived ERP components elicited in timing of the rat-MMN appearance exhibited a deviant effect (deviant > non-deviant) and a repetition effect (non-deviant > standard). Initially, the ERP waveform at the AC exhibited no MMN-like deflection morphologically like human MMN did, irrespective of its polarity. The ERP waveform has two notable negative components that appear to be elicited by the onset (on-response) and the falling edges (off-response) of tone stimulation. Since such on- and off-response have been reported as typical unit-response patterns of neurons in the primary AC (PAC)^52^, the bi-humped ERP waveform recorded from the AC may reflect population EPSPs (excitatory post-synaptic potentials) of neurons in the PAC. Of course, there is also a possibility that the polarity of the rat-MMN component could be reversed in recording from an electrode on the surface of the PAC, because the polarity of the rodents-MMN can change depending on electrode location or depth^37^. To examine this possibility, we measured the peak amplitude of a positive deflection (P80) between N30 and N130. The amplitude of P80, however, did not show a significant difference between deviant- and non-deviant tones, indicating that the P80 was not deviant-specific. This fact suggests that the MMN-like potential with a deviant-specific characteristic cannot be recorded at least from the surface of the PAC, regardless of the polarity of ERP components, at least, in the present experimental condition. Although previous studies on human being have indicated that the locus of the MMN generator should primarily be in the AC^21,53–60^, we failed to detect the MMN-like potential in the present study in which an electrode was placed on the surface of rat PAC. Alternatively there is a report that the MMN-like potential could not be measured epidurally over the dorsal portion of PAC^38^.

It is now unknown why the MMN-like potential cannot be recorded from the surface of the PAC. A recent study, however, reported that the local field potential (LFP) recorded in the PAC of awake mice exhibited a significantly enhanced response to the deviant tone, in comparison with the one to the non-deviant tone^61^. Though this fact suggests a possible involvement of the PAC in generating the MMN, the difference in LFP amplitude between deviant- and non-deviant tones appears very small at least within the PAC, in which the LFP trace to the deviant tone almost overlaps with the one to the non-deviant tone. It should be noted here that the recording method was different between Parras’s study and ours. They recorded LFPs intracortically through a fine needle electrode used for unit recording inserted in the PAC, while we recorded EEG activity epidurally via a relatively wide screw electrode allocated on the surface of the PAC. Therefore, our results seems not necessarily to contradict the finding of Parras’s study.

Pincze *et al*.^55^ suggested by using a cat model that the main locus of the MMN generator was the rostroventral part of the secondary auditory cortex. The ventral secondary auditory cortex (VSAC) is a structure just ventral to the PAC in rats. The electric potential at the recording electrode is proportional to the cosine of the angle between the recording surface and the current dipoles formed by electrical activity of the pyramidal neurons in the VSAC. Since it is assumed that the current dipoles in the VSAC were primarily formed in the normal direction against the brain surface by pyramidal neurons, the recording surface of the electrode placed on the dorsal PAC should be relatively highly angled compared with the angle of the current dipoles. This produces considerable attenuation of the VSAC-derived potential recorded at the surface of the PAC. However, the recording surface of the electrode placed on the PAC should be at 0 degrees from the current dipoles formed in the PAC, and relatively large potential is generated in the PAC as a modality-specific sensory response by auditory stimulation. Such large near-field potential derived from the PAC may primarily contribute to the potential recorded on the surface of the PAC, while the potential derived from the VSAC may be overwhelmed by the sensory-evoked large potential in the PAC.

Why can the rat-MMN be recorded from the frontal cortex when the direct MMN generator is located far from the FC? In human MMN studies, the frontal lobes have been shown to be another source of MMN in addition to the auditory cortex^19,62–65^. A recent clinical study reported that high-γ (80–150 Hz) activities recorded from the frontal cortex exhibits remarkable sensitivity to deviant and unpredictable events, whereas the activity derived from the temporal cortex is less sensitive to predictability of a deviant stimulus or the degree of periodicity of deviant event occurrence^66^. This result indicates that frontal and temporal cortices may have different function in signaling a deviation from background stimuli. In addition, several studies have proposed subcortical contribution to the MMN. The MMN-like potential elicited by deviant tones was found in the nonprimary polimodal area of the medial geniculate body of the thalamus in guinea pigs^38^. Patients with small thalamic infarctions exhibited a marked decrease in MMN-like neuromagnetic responses to deviant tones^67^. Further, Parras *et al*.^61^ recently demonstrated that the neuronal mismatch responses systemically increased from the inferior colliculus to the medial geniculate nucleus of the thalamus and the AC, and from lemniscal- to non-lemniscal divisions within each level^61^. These facts suggest that multiple brain regions are hierarchically involved in elicitation and/or modulation of the MMN generation and/or processing. With this respect, it is less likely that the MMN-like potential recorded from the FC is directly derived from the volume conduction of the electric source in the VSAC, because the recording surface of the electrode placed on the frontal cortex is estimated to be at about 90 degrees (cos 90° = 0) from the current dipoles formed in the VSAC. Therefore, the rat-MMN recorded from the FC may primarily be dependent on a relatively broad electric field formed by the activity of multiple thalamo-cortical circuits, although we do not rule out any other explanations with the given volume and structure differences between rat and human brains.

In addition to the reduced amplitude of MMN, EGF-treated rats exhibited the alteration in time-frequency responses of EEG recorded at the frontal cortex. The response pattern to the deviant tone in EGF-treated rats resembled the response pattern in control rats to the non-deviant tone, with the same probability of occurrence and the same pitch as the deviant tone. This suggests that EGF-treated rats may perceive the deviant tone as the less-deviant one, or as a less-changed event. Such an altered responsiveness of EEG activity to the deviant tone in the EGF group may also be suggested by the statistical result that a significant interaction was detected between groups (control vs. EGF) and stimulus type (deviant vs. non-deviant). Therefore, EGF-treated rats may have difficulty recognizing sound pitch differences as suggested in patients with schizophrenia^68^.

In our present study the rat-MMN to the standard tone was significantly smaller than the one to the non-deviant tone that was physically identical with the standard tone except the probability of occurrence (the repetition effect) in both EGF- and control groups. This fact indicates that the EGF-treated rats exhibited the normal repetition effect of the same tone on the amplitude of rat-MMN. However, a previous study reported that the repetition effect on the MMN was impaired in patients with schizophrenia, as well as the smaller MMN to the deviant tone, compared with the one in healthy controls^69^. If the EGF-treated rats can be an animal model of schizophrenia, this result appears contradictory to ours. Since their task condition was quite different from ours, we cannot directly compare their results with the present results, however. Of course, such a discrepancy may suggest a limitation of our animals as a model of schizophrenia. Further studies would be needed to address this problem.

In conclusion, this study demonstrates that perinatal administration of EGF reduces the MMN-like potential recorded at the FC after attaining maturation in rats. This MMN reduction in EGF-treated rats is partly due to reduced synchronization of EEG components evoked or invoked by tone stimulation across trials. Since the reduction of MMN amplitude^22–26^ and reduced gamma synchronization^70^ are consistently seen in patients with schizophrenia, the animals that received EGF injection during a neonatal period may be a promising neuro-developmental model of schizophrenia. Further studies should elucidate the pathogenesis of the MMN abnormality in EGF-treated animals to verify its relevancy to schizophrenia.

## Methods

### Animals

Male Sprague-Dawley (SD) rats (Japan SLC, Inc., Shizuoka, Japan) (n = 45) were used. Animals were housed in the Niigata University Animal Facility under a reversed 12-h light/dark cycle (8:00 am OFF and 20:00 pm ON), at constant temperature and humidity, with solid food and water available ad libitum. After surgery, solid foods were replaced into foamed soft food pellets (CMS sprout, Oriental Yeast Co., Itabashi Tokyo Japan) to enhance recovery. Two rats exhibited more than 10% weight loss and were excluded from EEG recording. All efforts were made to minimize animal suffering and to reduce the number of animals used. The treatment of these animals was in accordance with the local and international guidelines on the ethical use of laboratory animals. All procedures adopted in this study were approved by and conducted under the control of the Niigata University Animal Care and Use Committee.

### Preparation of neonatal EGF-treated rats

Each male newborn-littermate was pseudo-randomly assigned to either EGF-treated group or control group, so that both groups would be nearly equal size. In the EGF group recombinant human EGF (Higetsu-Shouyu, Chiba, Japan) dissolved in physiological saline was subcutaneously administered (875 ng/g) repeatedly on the nape to rat pups from postnatal day 2 to 10. Control littermates received injections of physiological saline at the same dose with EGF.

### Surgery and electrode placement

Rats (3–4 months old) were anaesthetized with pentobarbital (Somnopentyl; 65 mg/kg, i.p., Kyoritsu, Tokyo, Japan) and were positioned in a stereotaxic frame (SR-6M, Narishige) according to the atlas of Paxinos and Watson^71^. The wound margin was infiltrated with 2% lidocaine (Maruishi, Osaka, Japan). Miniature stainless steel bolts (1.0 mm diameter) as electrodes for recording cortical EEG were unilaterally screwed to the skull at the following points to contact the dura mater: the frontal cortex (FC), located at 2 mm anterior to bregma, 2 mm lateral to the midline not to damaging the sagittal sinus; and the auditory cortex (AC), 4.5 mm posterior to bregma, 8.0 mm lateral to the midline, 4.2 mm below the top-plane of the skull. Special attention was paid to minimize the injury of the temporalis muscle; a hemispheral incision was made along the muscle fibers with an radio knife (PROG-DS3M, Morita Tokyo Ltd, Saitama, Japan). In addition, the replacement with the soften food pellets warranted normal weight gain. Other bolts were screwed to the skull above the frontal sinus (10 mm anterior to bregma) as a reference and above the cerebellum (11 mm posterior to bregma) as a ground. All lead wires from the electrodes were soldered onto a miniature connector (socket) which was anchored to the cranium using dental cement. Rats received antibiotics (100 mg/kg cefmetazon, Daiichi-Sankyo Pharmaceutical Ltd., Tokyo Japan) after surgery. Recording began at least 14 days after recovery from surgery.

### Auditory stimulation

Rats were exposed to the following two different experimental conditions: In the oddball condition two different auditory stimuli were presented sequentially through a loud speaker at the stimulus onset asynchrony of 0.3 s. The auditory stimuli were 3000 Hz and 6000 Hz pure tones (85 dB SPL), both of which lasted 100 ms with a rise and decay time of 1 ms. These tones were presented in pseudo-random order so that the infrequent tone did not occur on consecutive trials. The total number of tone presentations was 2000. In 24 rats the probability of occurrence was set to 90% for 3000 Hz tone (standard tone), and 10% for 6000 Hz tone (deviant tone). The other 19 rats also underwent an additional condition, in which the probability of occurrence was reversed between the above two tones (90% for 6000 Hz, 10% for 3000 Hz). Because EEG signal could not be recorded in three rats, the data from these 3 rats were excluded from further analyses. Consequently, seven rats belonged to the control group, and nine rats did to the EGF group. In another condition (the many-standards condition) 10 different tones that varied in frequency were presented sequentially and randomly in the same way as the oddball condition described above. These stimuli were 1000, 1500, 2000, 3000, 4000, 5000, 6000, 7000, 7500, 8000 Hz pure tones. The probability of occurrence was uniformly 10% for every tone. This condition was executed to make the adaptation-independent comparison between deviant- and non-deviant stimuli, so that we could verify whether the MMN-like potential was larger than the deviant tone compared with the non-deviant tone having the same probability of occurrence as the deviant tone. This is a major criterion in identifying an ERP component like the human MMN-like potential^36,37^.

### Recording

Recording was executed in a dimly lit room. Each rat was placed in a transparent electrically-shielded plastic box (W36 cm; L18 cm; H30 cm) during recording. The signal from electrodes was fed by wire into a bioelectric amplifier (AB-611J, Nihon Kohden, Tokyo Japan) with a band-pass filter of 0.5–300 Hz via a miniature preamplifier (JH-440J, Nihon Kohden) directly attached to the socket on the rat’s head.

### Data analysis

EEG signal was sampled every 2 ms through the signal acquisition system (Micro1401-3, CED, Cambridge, UK). Tone-onset-locked average waveforms of event-related potentials were constructed from digitised EEG data for 300 ms, individually, for 3000 Hz and 6000 Hz tones in both the oddball and the many-standards conditions. In the oddball condition 200 out of all standard tones presented (1800) were randomly selected in order to match the number of tones available for constructing the average waveforms compared with the deviant tone.

To analyze the time-frequency characteristics of EEG activity to the deviant tone that exhibited the marked difference between EGF and control rats, we performed wavelet analysis on EEG data during a period around tone stimulation. The continuous wavelet transform (CWT) was applied to EEG data 100 ms before and 200 ms after each tone was presented. The CWT was calculated using Scilab ver.6.0 (Scilab Enterprises, Orsay Cedex, France) and its Wavelet Toolbox. The CWT was executed using the complex Morlet wavelet as a mother wavelet with the central frequency of 6. The scale parameter was set to 2^n^, where n ranged from 1 to 9 in increments of 1/32. The complex Morlet wavelet function can provide us information about magnitude (amplitude) and phase, independently.

Results in the text and figures are presented as the mean ± SEM. All statistical analyses were performed using SPSS Statics ver.21 (IBM, Armonk, USA), if not specified. Repeated measures analyses of variance were performed for factors of group (EGF, control), recording site (FC, AC), and stimulus type (deviant, non-deviant, standard). In all statistical tests, a *P*-value less than 0.05 was considered statistically significant. Bonferroni correction was used for multiple comparisons.

## Supplementary information

Supplementary figures
