# Supplementary material for: Neonatal exposure to an inflammatory cytokine, epidermal growth factor, results in the deficits of mismatch negativity in rats
Source: Sci Rep. 2019 May 16;9:7503. doi: 10.1038/s41598-019-43923-y (PMC6522493; doi:10.1038/s41598-019-43923-y)
Supplement: Supplementary file 1 — Supplementary figures [file 41598_2019_43923_MOESM1_ESM.pdf]

Supplementary Materials

Neonatal exposure to an inflammatory cytokine, epidermal growth factor, results in the deficits of mismatch negativity in rats

Eiichi Jodo, Hiroyoshi Inaba, Itaru Narihara, Hidekazu Sotoyama, Eiko Kitayama, Hirooki Yabe, Hisaaki Namba, Satoshi Eifuku, Hiroyuki Nawa

Fig S1. Grand average event-related potentials (ERPs) derived from the frontal cortex to deviant, non-deviant, and standard tones, the frequency of which was identical (6000 Hz).

Fig S2. Grand average event-related potentials (ERPs) derived from the auditory cortex to deviant, non-deviant, and standard tones, the frequency of which was identical (6000 Hz).

Fig S3. Mean EEG magnitudes during a baseline period for 100 ms before the start of tone presentation in individual frequency ranges (theta 4 –13 Hz, beta 14 –29 Hz, gamma 30 –80 Hz) in the frontal cortex (a) and the auditory cortex (b) are compared between control and EGF-treated rats.

Fig S4. Mean relative magnitudes of EEG responses during a period from 25 ms to 140 ms after the start of tone presentation in individual frequency ranges in the frontal cortex (a) and the auditory cortex (b) are compared between control and EGF-treated rats.

METHODS

All materials and methods were same as described in the main text, except the following auditory stimulation paradigm;

Auditory stimulation. Rats were exposed to the following two different experimental conditions: In the odd-ball condition two different auditory stimuli were presented sequentially through a loud speaker at the stimulus onset asynchrony of 0.3 s. The auditory stimuli were 3000 Hz and 6000 Hz pure tones (85 dB SPL), both of which lasted 100 ms with a rise and decay time of 1 ms. These tones were presented in pseudo-random order so that the infrequent tone did not occur on consecutive trials. The total number of tone presentations was 2000. In 19 rats the probability of occurrence was set to 90% for 3000 Hz tone (standard tone), and 10% for 6000 Hz tone (deviant tone). They also underwent an additional condition, in which the probability of occurrence was reversed between the above two tones (90% for 6000 Hz, 10% for 3000 Hz). Because EEG signal could not be recorded in three rats, the data from these 3 rats were excluded from further analyses. Consequently, seven rats belonged to the control group, and nine rats did to the EGF group. In another condition (the many-standards condition) 10 different tones that varied in frequency were presented sequentially and randomly in the same way as the odd-ball condition described above. These stimuli were 1000, 1500, 2000, 3000, 4000, 5000, 6000, 7000, 7500, 8000 Hz pure tones. The probability of occurrence was uniformly 10% for every tone. This condition was executed to make the adaptation-independent comparison between deviant- and non-deviant stimuli, so that we could verify whether the MMN-like potential was larger than the deviant tone compared with the non-deviant tone having the same probability of occurrence as the deviant tone. This is a major criterion in identifying an ERP component like the human MMN-like potential.

Supple Fig S1

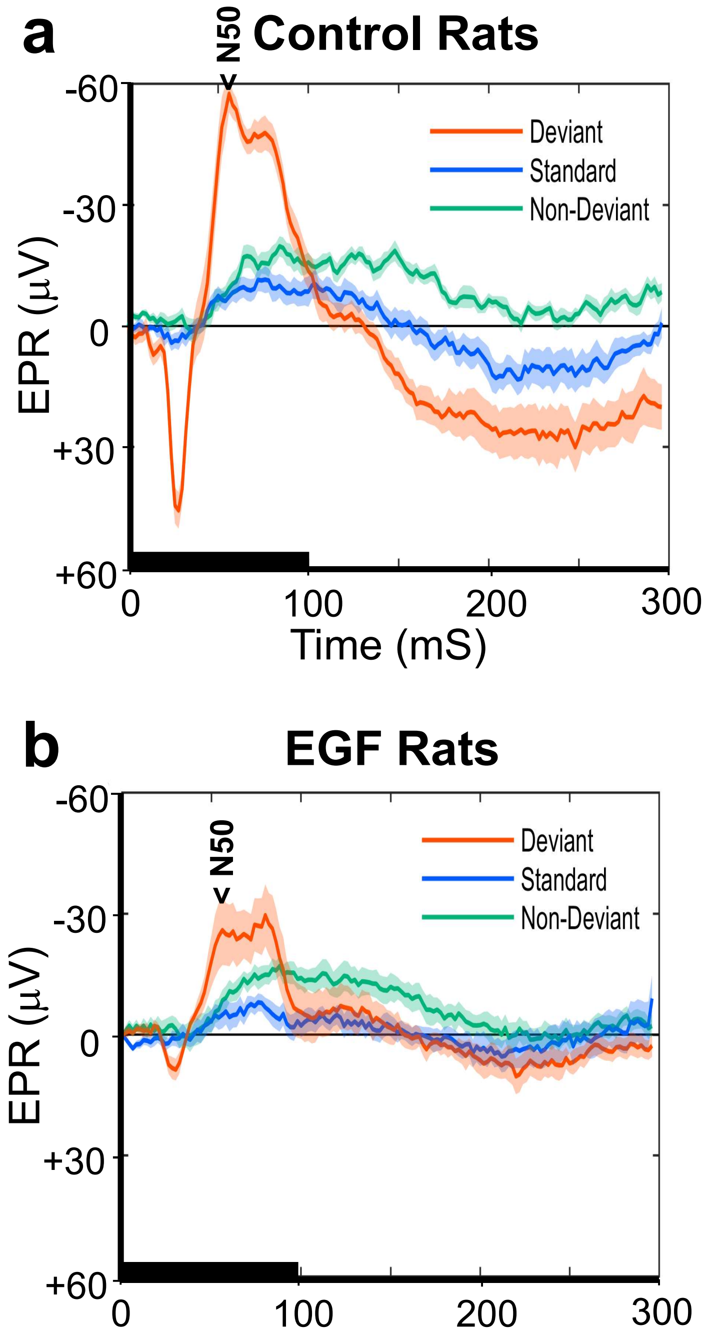

Fig. S1 Grand average event-related potentials (ERPs) derived from the frontal cortex to deviant, non-deviant, and standard tones, the frequency of which was identical (6000 Hz). ERP waveforms were recorded from an electrode on the frontal cortex in control (a)- and EGF (b)- treated rats and were grand averaged across subjects. The shaded area around each ERP trace denotes the mean ± standard error (SE). The tone stimulation is marked with a thick horizontal line lasting for 100 ms. Data were obtained from 7 control and 9 EGF-treated rats.

Supple Fig S2

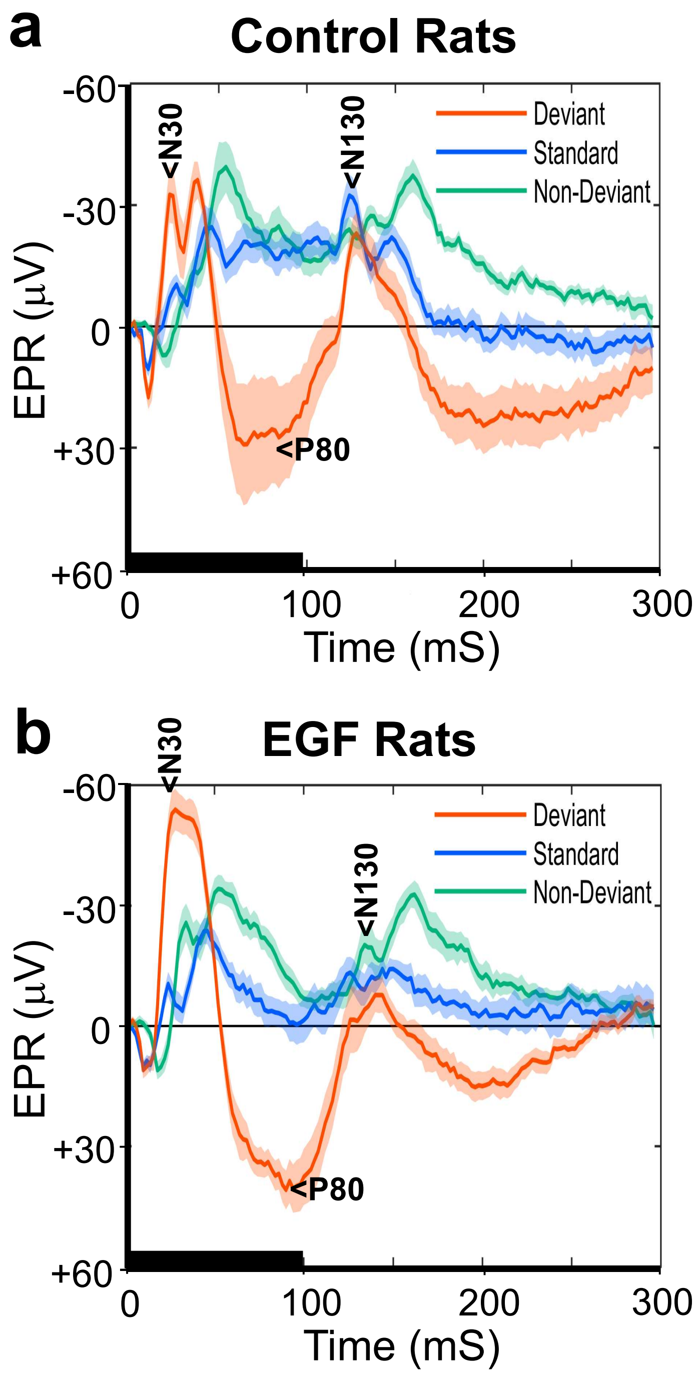

Fig. S2 Grand average event-related potentials (ERPs) derived from the auditory cortex to deviant, non-deviant, and standard tones, the frequency of which was identical (6000 Hz). ERP waveforms were recorded in the auditory cortex in control (a)- and EGF (b)-treated rats and were grand averaged across subjects. Other descriptions are the same as those depicted in Fig.S1.

Supple Fig S3

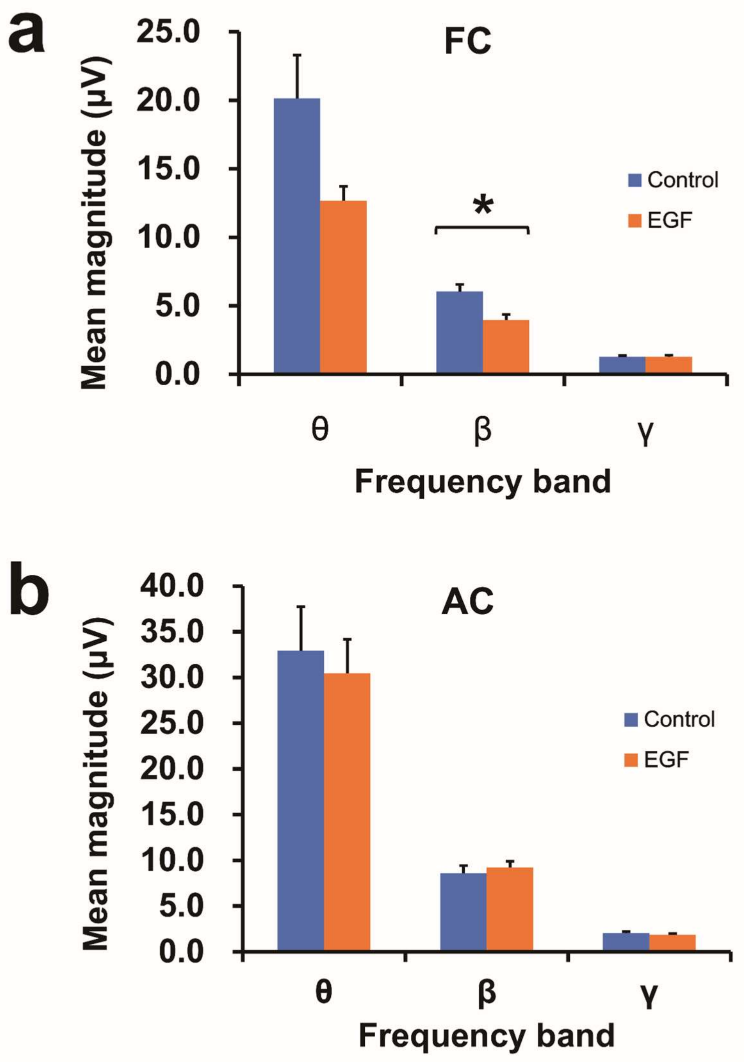

Fig. S3 Mean EEG magnitudes during a baseline period for 100 ms before the start of tone presentation in individual frequency ranges (theta 4 −13 Hz, beta 14 −29 Hz, gamma 30 −80 Hz) in the frontal cortex (a) and the auditory cortex (b) are compared between control and EGF-treated rats. Asterisks denote a significant difference between groups (*$p < 0.05$).

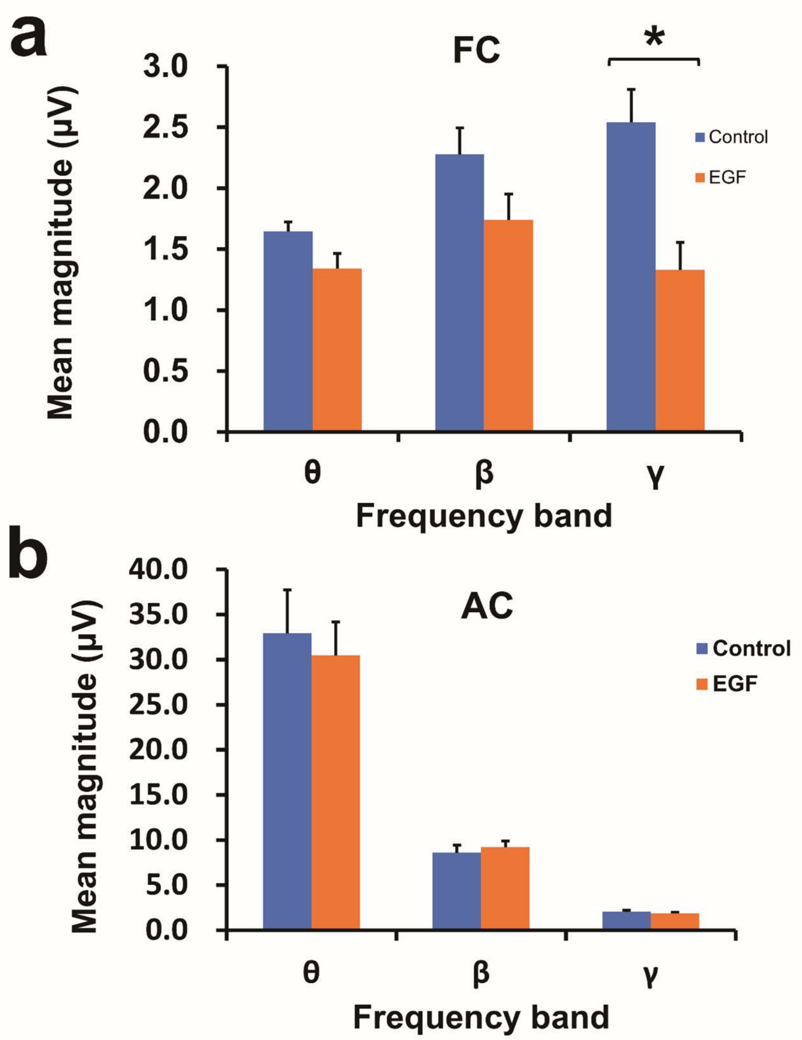

Fig. S4 Mean relative magnitudes of EEG responses during a period from 25 ms to 140 ms after the start of tone presentation in individual frequency ranges in the frontal cortex (a) and the auditory cortex (b) are compared between control and EGF-treated rats. The relative magnitude was obtained as a ratio of the average magnitude during a post-stimulation period (25 – 140 ms) to the average magnitude during a baseline period in individual frequency band. Other descriptions are the same as those depicted in Fig.S3.
